# Early-Onset Depression in Stroke Patients: Effects on Unfavorable Outcome 5 Years Post-stroke

**DOI:** 10.3389/fpsyt.2021.556981

**Published:** 2021-06-25

**Authors:** Ya-Ying Zeng, Meng-Xuan Wu, Sheng-Nan Zhou, Dan-Dan Geng, Lin Cheng, Kai-Li Fan, Xin Yu, Wen-Jie Tang, Jin-Cai He

**Affiliations:** ^1^Department of Neurology, The First Affiliated Hospital of Wenzhou Medical University, Wenzhou, China; ^2^First School of Clinical Medicine, Wenzhou Medical University, Wenzhou, China; ^3^School of Mental Health, Wenzhou Medical University, Wenzhou, China

**Keywords:** depression, post-stroke depression, disability, outcome, stroke

## Abstract

**Background:** Post-stroke depression (PSD) constitutes an essential complication of stroke and is associated with high-risk unfavorable outcome after stroke. The main objective of this prospective study was to determine the relationship between early-onset PSD (1 month after stroke) and functional outcomes 5 years after baseline enrollment.

**Methods:** Four hundred thirty-six patients who met the criteria were included in this study from October 2013 to February 2015. The follow-up time for each patient was ~5 years, with follow-up every 3 months. Patients received questionnaires including the 17-item Hamilton Depression Scale (HAMD), the Mini-Mental State Examination (MMSE), the National Institutes of Health Stroke Scale (NIHSS), the modified Rankin Scale (mRS), and the Barthel Index (BI).

**Results:** Of the 436 patients, 154 (35.3%) patients with the prevalence of PSD status at baseline, 26 (7.2%) patients with the prevalence of PSD status, and 73 (20.1%) had an unfavorable outcome 5 years after stroke. The odds ratio (OR) for unfavorable outcome at 5 years in the PSD group was ~2.2 relative to the non-PSD group after adjusting for potential risk factors [OR = 2.217, 95% confidence interval (CI) = 1.179–4.421, *P* = 0.015]. In the early-onset PSD group, HAMD scores were independently associated with 5-year unfavorable outcome rates (OR = 1.168, 95% CI = 1.015–1.345, *P* = 0.031).

**Conclusions:** Our findings indicate that early-onset PSD status in Chinese patients is an independent risk factor for unfavorable outcome 5 years after stroke, and that the severity of PSD is also related to unfavorable outcome.

## Introduction

Post-stroke depression (PSD) has been reported to be one of the most common complications in patients who experienced a stroke (1). According to a recent meta-analysis, the pooled frequency of PSD is ~25–36% (2, 3). Due to statistical and methodological differences, the cumulative incidence of PSD within the first 5 years following stroke is ~39–52% (4).

As an essential complication of stroke (2, 5), PSD seriously affects the recovery and unfavorable outcome of patients after stroke (6, 7). Previous articles have explained that PSD is linked to clinical and functional outcomes. It is associated with high-risk disability and a low quality of life (QoL) in patients with stroke (8–10). PSD will reduce social interactions and reduce the success rate of rehabilitation (9, 11, 12). Most of these studies have limitations, including short follow-up times and small sample sizes. A paucity of well-designed studies with sufficiently large sample sizes has examined the long-term prognosis of PSD 5 years after stroke. Only one study by Ayerbe et al. elaborated on the association between depression 3 months after stroke and disability at 5 years after stroke from the South London Stroke Register using the Hospital Anxiety and Depression Scale (HADS), Glasgow Coma Scale (GCS), and Barthel Index (BI), but no group of studies reported such a long follow-up of patients with early-onset depression (1 month) and disability using the modified Rankin Scale (mRS) and Hamilton Depression Scale (HAMD). The relationship between the severity of depression and disability 5 years after stroke has not been elaborated (13). Therefore, research exploring PSD and the long-term prognosis and functional rehabilitation is critical and will also have implications for clinical treatment.

Many medical and social factors are considered the basis of the relationship between depression and unfavorable outcome, including diabetes, smoking, cognitive impairment, poor socialization, and negative perception of coping strategies. However, the specific mechanism underlying the association between PSD and unfavorable outcome is unclear. A better understanding of the relationship between depression and unfavorable outcome in clinical research will provide opportunities for research into specific mechanisms.

The objectives of this study were to (1) assess the incidence of depression and unfavorable outcome 5 years after stroke, (2) determine the relationship between early-onset PSD (1 month after stroke) and functional outcomes 5 years after baseline enrollment, and (3) examine the association between depression severity and functional outcomes in patients who experienced depression within the first month after stroke.

## Materials and Methods

### Study Design

We performed an analysis of prospectively collected data from October 2013 to February 2015 from patients treated in the stroke unit of the First Affiliated Hospital of Wenzhou Medical University.

The criteria for selecting the subjects were as follows: (1) age between 18 and 80 years and (2) diagnosed with computerized tomography (CT) or magnetic resonance imaging (MRI) at the time of admission.

The exclusion criteria were as follows: (1) transient ischemic attack; (2) inability to be assessed for severe aphasia or dysarthria or visual or auditory impairment; (3) any central nervous system disease, such as dementia, severe cognitive impairment, or Parkinson's disease; (4) a history of major depression or other psychiatric disorders; (5) severe hepatic or renal diseases; and (6) refusal to be assessed.

After applying the inclusion and exclusion criteria, 436 hospitalized patients met the criteria. These 436 patients were followed and evaluated 1 month after they were discharged from the hospital. Due to a loss to follow-up, lack of disability, and good physical recovery at 1 month, 436 patients were included in this prospective study. We started the prospective study at 1 month after discharge. Among these 436 patients, 154 had PSD status, and 209 did not have PSD status.

All participants provided written informed consent. This study was approved by the Ethics Committee of the First Affiliated Hospital of Wenzhou Medical University and conformed to the Declaration of Helsinki.

### Assessments

A well-trained neurologist collected demographic data, including sex, age, body mass index (BMI), years of schooling, and smoking status and alcohol drinking history. Clinical data collected included a history of hypertension, diabetes, hyperlipidemia, coronary artery disease (CAD), and stroke. Cognitive function was assessed using the Mini-Mental State Examination (MMSE). CT or MRI was performed within 72 h after admission. The location of the ischemic lesion on the MRI or CT scan was determined by qualified radiologists who were blinded to the patients' psychiatric diagnoses.

### Depression

Neurologists and psychiatrists received psychiatric training. The diagnosis of depression was consistent with the Diagnostic and Statistical Manual of Mental Disorders, Fourth Edition (DSM-IV) criteria for depressive symptoms (HAMD score ≥7) (14, 15).

### Functional Status

Unfavorable outcome was assessed with the mRS and the BI at baseline and the 5-year follow-up (16, 17). A previous study defined an unfavorable outcome (disability) as an mRS score >2 (18). Therefore, we specified the mRS as a dichotomous variable with 0–2 points indicating favorable outcome and 3–6 representing unfavorable outcome. The BI assessment included self-care (feeding, grooming, bathing, dressing, bowel and bladder care, and toilet use) and mobility (ambulation, transfers, and stair climbing) (19).

### Stroke Severity

We used the National Institutes of Health Stroke Scale (NIHSS) to assess the severity of stroke within 24 h of admission (20). Stroke severity was evaluated at admission with the NIHSS by trained neurologists, which includes assessments of consciousness, vision, speech, and sensory and motor functions.

### Follow-Up

Follow-up was performed at the outpatient clinic or by phone every 3 months. The mean time from the index stroke to final follow-up was 5 years (range 50–66 months). All patients were followed by a face-to-face interview at baseline. Follow-up at 5 years was performed by a telephone survey or face-to-face interview, depending on the capacity of the patient to come to the hospital. Due to a loss of telephone contact, unwillingness to continue participating in the study, and other reasons, 73 people were lost to follow-up, with a release rate of 16.7%, and 363 people completed the final follow-up. Finally, face-to-face interviews were performed for 38 of 363 patients, and telephone interviews were available for all 325 patients at the 5-year follow-up.

### Statistical Analysis

SPSS software was used to statistically analyze patients' baseline information and risk factors. Categorical variables were tested with the chi-square or Fisher's exact-test and are shown as percentage. Continuous variables are presented as mean ± standard deviation (SD) or median and interquartile range (IQR), depending on whether they were normally distributed, and were compared using Student's *t*-test or the Mann–Whitney-test, as appropriate.

A multivariate logistic regression analysis was performed to assess whether early-onset PSD status might be an independent predictor of unfavorable outcome 5 years after stroke and included variables related to 5-year functional outcomes identified in the bivariate analysis, as well as variables determined based on previous clinical judgment. Model 1 was adjusted for age, sex, BMI, history of smoking and alcohol drinking, and early-onset PSD. Model 2 was adjusted for covariates in model 1 and further adjusted for education years, baseline NIHSS score, and baseline MMSE score. Model 3 was adjusted for covariates in model 2 plus medical history (hypertension, diabetes mellitus, hyperlipidemia, coronary heart disease, and stroke).

By repeating the above binary and multivariate analyses, we assessed the effect of depression severity on the outcomes 5 years after stroke exclusively in patients with depression (*n* = 154).

All analyses were carried out using SPSS, version 19. *P*-values < 0.05 were considered statistically significant. Patients with missing data were excluded from the analysis.

## Results

**Figure 1:**
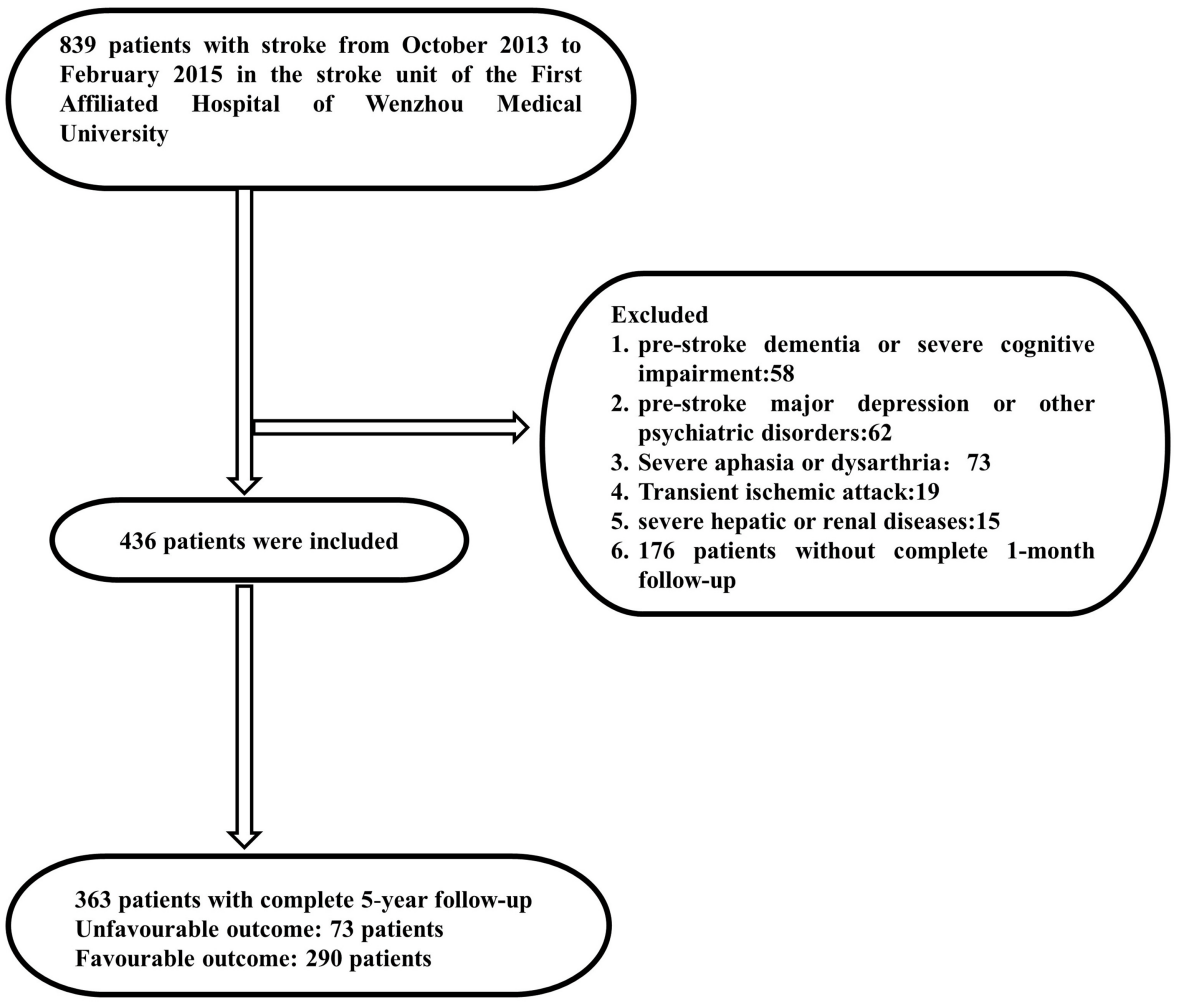
Study flow diagram.

From February 2013 to July 2015, 436 of the 839 hospitalized patients who met the criteria completed a face-to-face questionnaire 1 month after discharge. Ultimately, we included these 436 patients in this prospective study. The mean time from the index stroke to final follow-up was 50–66 months, and 363 people completed the final follow-up. A flowchart of the study is shown in Figure 1. Among patients who completed depression interviews, 26/363 (7.2%) patients presented with PSD status, including 5/38 (13.2%) who completed face-to-face interviews and 21/345 (6.1%) who completed telephone interviews.

Of the 363 patients who completed the 5-year follow-up and were included in the study, 154 had PSD status, and 209 were not diagnosed with PSD status. In the PSD group, patients had an average age of 61.9 years, and the median NIHSS score was 3.5 (IQR, 2–6). In the non-PSD group, the average age was 62.7 years, and the median NIHSS score was 2 (IQR, 1–4). Compared with non-PSD patients, patients with PSD had poor cognitive function (MMSE score: 22.5 vs. 21.1, *P* = 0,019), lower BI scores [95 (65–100) vs. 60 (40–90), *P* < 0.001], and lower mRS scores [1 (1–3) vs. 2 (1–4), *P* < 0.001]. The percentage of unfavorable outcome in the PSD group was much higher than that in the non-PSD group (29.2 vs. 13.4%, *P* < 0.001) (Table 1).

**Table 1:** Baseline characteristics of patients with acute ischemic stroke (*N* = 363).

**Group**	**Non-PSD**	**PSD**	*P*-value^*^
*N*	209	154	
Age (y), mean ± SD	62.7 ± 10.5	61.9 ± 12.3	0.554
Gender, male *n* (%)	69 (33.2%)	62 (41.1%)	0.125
BMI (kg/m^2^)	24.3 ± 9.2	24.1 ± 4.1	0.824
Education (years)	4.3 ± 3.9	4.0 ± 3.5	0.413
**Lesion location**			
Frontal lobe, *n* (%)	31 (17.5%)	24 (18.0%)	0.904
Parietal lobe, *n* (%)	17 (9.6%)	11 (8.3%)	0.685
Temporal lobe, *n* (%)	12 (6.8%)	14 (10.5%)	0.239
Occipital lobe, *n* (%)	14 (4.5%)	10 (7.5%)	0.899
Basal ganglia, *n* (%)	78 (44.1%)	75 (56.4%)	0.032^*^
Cerebellum, *n* (%)	18 (10.2%)	16 (12.0%)	0.604
Midbrain, *n* (%)	20 (11.3%)	13 (9.8%)	0.667
Pons, *n* (%)	29 (16.4%)	21 (15.8%)	0.888
Medulla, *n* (%)	23 (13.0%)	40 (12.9%)	0.956
History of drinking, *n* (%)	78 (39.2%)	44 (30.6%)	0.099
History of smoking, *n* (%)	66 (31.7%)	43 (28.7%)	0.534
History of hypertension, *n* (%)	150 (72.5%)	111 (72.5%)	0.986
History of diabetes, *n* (%)	42 (20.4%)	39 (25.5%)	0.253
History of hyperlipidemia, *n* (%)	17 (8.3%)	17 (11.1%)	0.368
Coronary artery disease, *n* (%)	18 (8.8%)	8 (5.3%)	0.206
History of a previous stroke, *n* (%)	19 (9.2)	17 (11.1)	0.556
MMSE, mean ± SD	22.5 ± 5.5	21.1 ± 5.5	0.019^*^
HAMD, median (IQR)	3 (1–6)	6 (3–9)	0.001^*^
NIHSS, median (IQR)	2 (1–4)	3.5 (1–6)	<0.001^*^
mRS, median (IQR)	1 (1–3)	2 (1–4)	<0.001^*^
BI, median (IQR)	95 (65–100)	60 (40–90)	<0.001^*^
Unfavorable outcome at baseline, *n* (%)	32 (11.3%)	51 (33.3%)	<0.001^*^
Unfavorable outcome at 5-years, *n* (%)	28 (13.4%)	45 (29.2%)	<0.001^*^

*BMI, body mass index; NIHSS, National Institutes of Health Stroke Scale; mRS, modified Rankin Scale; MMSE, mini mental state examination; BI, modified Barthel Index; IQR, interquartile range; SD, standard deviation; PSD, Post-stroke depression.*

* *p < 0.005*.

### Relations Between Early-Onset Depression After Stroke and Unfavorable Outcome at the 5-Year Follow-Up

At 50–66 months post-stroke, 73 (20.1%) patients had an unfavorable outcome at the 5-year follow-up. Compared with patients without disabilities, patients with disabilities were older [67.5 (60.8–72.0) vs. 64.0 (54.0–70.0), *P* = 0.003], had an increased stroke severity [NIHSS, 4 (2–6) vs. 3 (1–4), *P* < 0.001], and had decreased cognition [MMSE, 21.0 (16.6–24.0) vs. 23.0 (19.0–27.0), *P* = 0.002]. In addition, they were more likely to have a history of hypertension (82.2 vs. 70.0%, *P* = 0.038) (Table 2).

**Table 2:** Baseline characteristics of patients with unfavorable outcome and favorable outcome (*N* = 363).

**Group**	**Favorable outcome**	**Unfavorable outcome**	***P*-value**
*N*	290	73	
Age (y), median (IQR)	64.0 (54.0–70.0)	67.5 (60.8–72.0)	0.002^*^
Gender, male *n* (%)	181 (63.3%)	47 (64.4%)	0.862
BMI (kg/m^2^)	24.40 ± 8.19	23.59 ± 3.70	0.429
Education (years)	4.42 ± 3.86	3.32 ± 3.16	0.012^*^
**Lesion location**			
Frontal lobe, *n* (%)	44 (17.8%)	11 (20.0%)	0.948
Parietal lobe, *n* (%)	23 (9.3%)	5 (7.9%)	0.734
Temporal lobe, *n* (%)	22 (8.9%)	4 (6.3%)	0.513
Occipital lobe, *n* (%)	21 (8.5%)	3 (4.8%)	0.321
Basal ganglia, *n* (%)	121 (49.0%)	32 (50.8%)	0.798
Cerebellum, *n* (%)	24 (9.7%)	10 (15.9%)	0.163
Midbrain, *n* (%)	28 (11.3%)	5 (7.9%)	0.581
Pons, *n* (%)	39 (15.8%)	11 (17.5%)	0.748
Medulla, *n* (%)	32 (13.0%)	8 (12.7%)	0.957
History of drinking, *n* (%)	97 (35.5%)	25 (35.6%)	0.977
History of smoking, *n* (%)	93 (32.6%)	16 (21.9%)	0.076
History of hypertension, *n* (%)	201 (70.0%)	60 (82.2%)	0.038^*^
History of diabetes, *n* (%)	63 (22.0%)	18 (24.7%)	0.631
History of hyperlipidemia, *n* (%)	32 (11.2%)	2 (2.7%)	0.027^*^
Coronary artery disease, *n* (%)	17 (6.0%)	9 (13.9%)	0.063
History of a previous stroke, *n* (%)	27 (9.4%)	9 (12.3%)	0.463
PSD (at baseline)	109 (37.6%)	45 (62.6%)	<0.001
MMSE, median (IQR)	23.0 (19.0–27.0)	21.0 (16.6–24.0)	0.002^*^
HAMD, median (IQR)	4 (2–7)	5 (3–9)	<0.001^*^
NIHSS, median (IQR)	3 (1–4)	4 (2–6)	<0.001^*^

*BMI, body mass index; NIHSS, National Institutes of Health Stroke Scale; MMSE, mini mental state examination; BI, modified Barthel Index; IQR, interquartile range; SD, standard deviation; PSD, Post-stroke depression.*

* *p < 0.005*.

In the multivariate logistic regression analysis, PSD was significantly associated with a high risk of unfavorable outcome 5 years after stroke after adjusting for age, sex, BMI, and history of smoking and alcohol drinking [odds ratio (OR) = 2.877, 95% confidence interval (CI) = 1.609–5.143, *P* < 0.001, Table 3, model 1]. After adjustment for covariates from model 1 and further adjustment for education years, baseline NIHSS score, baseline MMSE, score and medical history (coronary heart disease, diabetes mellitus, hyperlipidemia, hypertension, and stroke), PSD status remained significantly independently associated with unfavorable outcome (model 2: OR = 2.012, 95% CI = 1.017–3.084, *P* = 0.030; model 3: OR = 2.217, 95% CI = 1.179–4.421, *P* = 0.015). In addition, in model 3, the baseline NIHSS score was independently associated with unfavorable outcome at 5 years (OR = 1.177, 95% CI = 1.050–1.320, *P* = 0.005).

**Table 3:** Multivariate adjusted odds ratios for the association between early-onset PSD and unfavorable outcome at 5-years.

		**OR**	**(95%CI)**	*P*-value^*^
**Model 1**	**Non-PSD**	**Reference**		
	Early-onset PSD	2.877	1.609–5.143	<0.001^*^
**Model 2**	**Non-PSD**	**Reference**		
	Early-onset PSD	2.012	1.017–3.084	0.030^*^
**Model 3**	**Non-PSD**	**Reference**		
	Early-onset PSD	2.279	1.179–4.421	0.015^*^

*OR, odds radio; CI, confidence level; PSD, post-stroke depression.*

*Model 1: adjusted for age, sex, body mass index, history of smoking, and alcohol drinking.*

*Model 2: adjusted for covariates from Model 1 and further adjusted for education years, baseline NIHSS score, baseline MMSE score.*

*Model 3: adjusted for covariates from Model 2 and further adjusted for medical history (coronary heart disease, diabetes mellitus, hyperlipidemia, hypertension, stroke).*

* *p < 0.005*.

### Depression Severity and Unfavorable Outcome at 5 Years After Stroke

Table 4 shows the baseline characteristics of the early-onset PSD group at baseline (*n* = 154). Patients in the PSD group who had an unfavorable outcome 5 years after stroke were older (65.8 vs. 60.3 years, *P* = 0.011) and had a more severe stroke [NIHSS score of 4 (2–6.5) vs. 3 (2–5), *P* = 0.053] than patients without an unfavorable outcome.

**Table 4:** Baseline characteristics of early-onset PSD group (*n* = 154).

**Group *N***	**Favorable outcome 107**	**Unfavorable outcome 47**	*P*-value^*^
Age, mean ± SD	60.3 ± 13.2	65.8 ± 8.7	0.011^*^
Gender, male *n* (%)	59 (55.7%)	30 (66.7%)	0.209
BMI (kg/m^2^), mean ± SD	24.4 ± 4.2	23.5 ± 3.8	0.619
Education (years), mean ± SD	4.3 ± 3.6	3.2 ± 3.0	0.896
**Lesion location**			
Frontal lobe, *n* (%)	17 (18.5%)	7 (17.1%)	0.846
Parietal lobe, *n* (%)	9 (9,8%)	2 (4.9%)	0.502
Temporal lobe, *n* (%)	12 (13.3%)	2 (4.9%)	0.225
Occipital lobe, *n* (%)	8 (8.7%)	2 (4.9%)	0.723
Basal ganglia, *n* (%)	52 (68%)	24 (58.5%)	0.739
Cerebellum, *n* (%)	10 (10.9%)	6 (14.6%)	0.538
Midbrain, *n* (%)	10 (10.9%)	3 (7.3%)	0.754
Pons, *n* (%)	14 (15.2%)	7 (17.1%)	0.800
Medulla, *n* (%)	12 (13.0%)	5 (12.2%)	0.892
History of drinking, *n* (%)	31 (30.4%)	13 (31.0%)	0.947
History of smoking, *n* (%)	35 (33.3%)	8 (17.8%)	0.054
History of hypertension, *n* (%)	74 (68.5%)	37 (82.2%)	0.084
History of diabetes, *n* (%)	25 (23.1%)	14 (31.1%)	0.303
History of hyperlipidemia, *n* (%)	17 (15.7%)	0	0.005
Coronary artery disease, *n* (%)	5 (4.7%)	3 (6.7%)	0.615
History of a previous stroke, *n* (%)	12 (11.1%)	5 (11.1%)	1.0
MMSE, mean ± SD	21.6 ± 5.4	20.0 ± 5.6	0.213
HAMD, mean ± SD	6.8 ± 4.4	7.1 ± 4.3	0.701
HAMD 1 month, median (IQR)	9.0 (8.0–11.0)	11.0 (8.5–12.5)	0.017^*^
mRS, median (IQR)	2 (1–3)	3 (2.25–4)	<0.001^*^
ADL, median (IQR)	80 (47.5–95)	25 (40–55)	<0.001^*^
NIHSS, median (IQR)	3 (2–5)	4 (2–6.5)	0.053

*BMI, body mass index; NIHSS, National Institutes of Health Stroke Scale; mRS, modified Rankin Scale; MMSE, mini mental state examination; BI, modified Barthel Index; IQR, interquartile range; SD, standard deviation; PSD, Post-stroke depression.*

* *p < 0.005*.

The unfavorable outcome group scored significantly higher on the HAMD [9.0 (8.0–11.0) vs. 11 (8.5–12.5), *P* = 0.017] and mRS [3 (2.25–4) vs. 2 (1–3), *P* < 0.001)] at baseline than the favorable outcome group.

The results of the multivariate analysis of the early-onset PSD group are presented in Table 5. In model 1, we included age, sex, BMI, and the HAMD score and found that the HAMD score at baseline was independently associated with the 5-year unfavorable outcome in the PSD group (model 1: OR = 1.126, 95% CI = 1.008–1.257, *P* = 0.036). Model 2 was adjusted for covariates from model 1 and further adjusted for education years, baseline NIHSS score, baseline MMSE score, and history of smoking and alcohol drinking. Model 3 was further adjusted for medical history (coronary heart disease, diabetes mellitus, hyperlipidemia, hypertension, and stroke) and the covariates included in model 2. The HAMD score remained significantly independently associated with unfavorable outcome (model 2: OR = 1.123, 95% CI = 0.989–1.277, *P* = 0.074; model 3: OR = 1.168, 95% CI = 1.015–1.345, *P* = 0.031).

**Table 5:** Multivariate adjusted odds ratios for the association between early-onset depression severity and unfavorable outcome at 5-year follow-up.

		**OR**	**(95%CI)**	*P-*value^*^
Model 1	Depression severity, HAMD score	1.126	1.008–1.257	0.036^*^
Model 2	Depression severity, HAMD score	1.123	0.989–1.277	0.074
Model 3	Depression severity, HAMD score	1.168	1.015–1.345	0.031^*^

*OR, odds radio; CI, confidence level; PSD, post-stroke depression.*

*Model 1: adjusted for age, sex, body mass index.*

*Model 2: adjusted for covariates from Model 1 and further adjusted for education years, baseline NIHSS score, baseline MMSE score, history of smoking and alcohol drinking.*

*Model 3: adjusted for covariates from Model 2 and further adjusted for and medical history (coronary heart disease, diabetes mellitus, hyperlipidemia, hypertension, stroke).*

* *p < 0.005*.

## Discussion

To the best of our knowledge, this large-sample study in China is the first to assess early-onset depression and unfavorable function outcome 5 years after stroke. Based on the present study, early-onset depression in the acute phase with stroke was significantly associated with a high risk of unfavorable outcome at 5-year follow-up, even after adjustment for several potential confounders. Among patients with early-onset PSD status, the baseline depression severity remained significantly independently associated with unfavorable outcome at 5 years after stroke. In addition, 7.2% of patients with stroke developed PSD status over the 5-year follow-up period.

Among patients who were assessed at 1 month after stroke, the prevalence of using the HAMD assessment approach was 35.3%, which is consistent with the estimates of PSD reported by Ayerbe et al., who documented a prevalence of 32.8% at 1 month after stroke (13). The current findings are also consistent with previous findings that the prevalence of depression among patients with stroke in Western and East Asian populations ranges from 30 to 50% (3, 21).

However, the incidence of PSD status after 5 years in our study was only 7.2%, a value that is much lower than that reported in another study using the HADS (13). The other study indicates that the incidence of depression varies according to the assessment method. Self-rated Geriatric Depression Scale (GDS) results in the highest rate of depressive symptoms (39.8%) prevalence after 5 years of stroke, and the incidence rate of depression symptoms assessed using the Cornell scale was 12.4%. DSM criteria produced the lowest rate of depressive symptoms (0.8%) (22). The discrepancy in our study might be explained by the measurement tools to assess depression. Most patients participated in telephone interviews, and the stigma of mental illness, especially in Chinese society, is such that they are reluctant to discuss symptoms of depression (23).

In our study, the incidence of poor outcomes 5 years after stroke was 20.1%, which was slightly lower than the value reported in previous studies. Chausson et al. defined an mRS score ≥3 as unfavorable outcome, and the prevalence of unfavorable outcome was 88/262 (33.6%) at 5 years after stroke (24). Geng et al. defined an mRS score >3 as a poor prognosis, indicating that the incidence of a poor prognosis 18 months after stroke was 29.8% (25). Li et al. defined an mRS score ≥2 as unfavorable outcome and found a prevalence of 36.0% 5 years later (26). We excluded patients who were over 80 years old and had more severe strokes at baseline and used a stricter definition of unfavorable outcome, which might have led us to underestimate the actual incidence of poor outcomes at 5 years.

Previous articles have reported that the diagnosis of stroke in the acute phase of PSD might independently predict the high disability rate of general stroke survivors (10, 27). Other studies have not found a correlation between depression and functional outcomes after stroke (28, 29). The time after stroke is important when studying the relationship between depression and unfavorable outcome. Most studies focus on short-term unfavorable outcome after stroke, but few articles focus on long-term results after stroke. To our knowledge, this study is the first to prospectively investigate the associations of early-onset PSD with unfavorable outcome 5 years after stroke in China. Our study confirms that early-onset PSD is correlated with unfavorable outcome after adjusting for confounders, which may be explained by the fact that patients with early-onset PSD had low activity levels and severe stroke (30–32). Compared with non-PSD patients, patients with PSD at admission had higher NIHSS and mRS scores, which also highlights the effect of PSD on patients' disabilities.

Notably, when we included only patients who had been depressed at baseline, the severity of depression was also related to unfavorable outcome 5 years after stroke. After adjustment for several potential confounders, the severity of depression was still a predictor of the 5-year unfavorable outcome incidence. Moreover, cognition (MMSE score) was significantly associated with unfavorable outcome in the univariate analysis. After adjustment for several potential confounders, it was not an independent predictor of unfavorable outcome at 5 years after stroke.

Possible explanations for the association between PSD and unfavorable outcome in our study may be that a depressed mood interferes with processes of learning, which are thought to underlie recovery from stroke (33, 34). Patients with depression and stroke have higher NIHSS scores at admission and a lack of motivation to participate in rehabilitation (35). Another possible explanation is that neuroplasticity is impaired in patients with depression and its mechanism is similar to rehabilitation (36, 37).

Other potential research limitations also exist. First, patients with aphasia, patients with severe stroke and high NIHSS scores, or patients whose depression was severe and could not be assessed were excluded, which may generate some biases in the results. Second, the sample size of patients with depression and unfavorable outcome was small, and a larger sample size would have provided greater sensitivity for the detection of the association between depression severity and unfavorable outcome. Third, the 5-year assessment of depression in most patients was a telephone follow-up, which may underestimate the incidence of depression 5 years after stroke. Instead, all 1-month depression assessments were conducted by face-to-face interviews, and the main results of the study were credible. Further studies are needed to determine the reliability of telephone interviews in assessing the depression status using the HAMD during long-term follow-up after stroke. Moreover, we did not analyze the relationship between early-onset PSD status and mortality due to the limited sample size in this study.

Despite the limitations described above, this extensive study is the first of Chinese patients with early depression and unfavorable function outcome 5 years after stroke. This study provides new information on functional ability and the prevalence of depression and unfavorable function among stroke survivors in China. We also found that patients with early-onset PSD status more frequently suffered from an unfavorable function and depression severity was related to unfavorable function 5 years after stroke. Therefore, early-onset depression still predicts unfavorable function in long-term follow-up and requires more clinical attention at an early stage.

## Data Availability Statement

The data analyzed in this study is subject to the following licenses/restrictions: The authors do not have permission to share data. Requests to access these datasets should be directed to hjc@wmu.edu.cn.

## Ethics Statement

All participants provided written informed consent. This study was approved by the Ethics Committee of the First Affiliated Hospital of Wenzhou Medical University, and conformed to the Helsinki Declaration.

## Author Contributions

Y-YZ and J-CH designed the study. Y-YZ and M-XW interpreted the data. Y-YZ wrote the manuscript. S-NZ, D-DG, and LC did the statistical analyses. Y-YZ, M-XW, LC, S-NZ, and K-LF screened and extracted the data. XY and W-JT supervised the study. All authors have made an intellectual contribution to the manuscript and approved the submission.

## Conflict of Interest

The authors declare that the research was conducted in the absence of any commercial or financial relationships that could be construed as a potential conflict of interest.

## Publisher's Note

All claims expressed in this article are solely those of the authors and do not necessarily represent those of their affiliated organizations, or those of the publisher, the editors and the reviewers. Any product that may be evaluated in this article, or claim that may be made by its manufacturer, is not guaranteed or endorsed by the publisher.

## Abbreviations

PSD: post-stroke depression
QoL: quality of life
HADS: Hospital Anxiety and Depression Scale
GCS: Glasgow Coma Scale
BI: Barthel Index
DSM-IV, Diagnostic and Statistical Manual of Mental Disorders: Fourth Edition
MMSE: Mini-Mental State Examination
mRS: modified Rankin Scale
NIHSS: National Institutes of Health Stroke Scale.
