# Corrigendum: Early-Onset Depression in Stroke Patients: Effects on Unfavorable Outcome 5 Years Post-stroke

**DOI:** 10.3389/fpsyt.2021.732437

**Published:** 2021-07-29

**Authors:** Ya-Ying Zeng, Meng-Xuan Wu, Sheng-Nan Zhou, Dan-Dan Geng, Lin Cheng, Kai-Li Fan, Xin Yu, Wen-Jie Tang, Jin-Cai He

**Affiliations:** ^1^Department of Neurology, The First Affiliated Hospital of Wenzhou Medical University, Wenzhou, China; ^2^First School of Clinical Medicine, Wenzhou Medical University, Wenzhou, China; ^3^School of Mental Health, Wenzhou Medical University, Wenzhou, China

**Keywords:** depression, post-stroke depression, disability, outcome, stroke

In the original article, there was an error in the order of authors. The correct author list is Ya-Ying Zeng^1, 2†^, Meng-Xuan Wu^3†^, Sheng-Nan Zhou^1†^, Dan-Dan Geng^1, 2^, Lin Cheng^1, 2^, Kai-Li Fan^2^, Xin Yu^2*^, Wen-Jie Tang^1, 2*^ and Jin-Cai He^1*^

The authors apologize for this error and state that this does not change the scientific conclusions of the article in any way. The original article has been updated.

## Publisher's Note

All claims expressed in this article are solely those of the authors and do not necessarily represent those of their affiliated organizations, or those of the publisher, the editors and the reviewers. Any product that may be evaluated in this article, or claim that may be made by its manufacturer, is not guaranteed or endorsed by the publisher.

